# Longitudinal blood pressure and body mass index in South African adolescents

**DOI:** 10.3389/fped.2025.1643812

**Published:** 2025-09-08

**Authors:** Elandi van Niekerk, Ruan Kruger, Caroline Sedumedi, Sanette J. Brits, Makama Andries Monyeki

**Affiliations:** ^1^ Physical Activity, Sport and Recreation (PhASRec), North-West University, Potchefstroom, South Africa; ^2^ Hypertension in Africa Research Team (HART), North-West University, Potchefstroom, South Africa; ^3^ Medical Research Council: Research Unit for Hypertension and Cardiovascular Disease, North-West University, Potchefstroom, South Africa

**Keywords:** adolescent, blood pressure, body mass index, diastolic, mean arterial, mid, systolic

## Abstract

**Aim:**

Blood pressure (BP) is known to be affected by body mass index (BMI) from an early age, but research in South African youth is scarce. We assessed longitudinal trends and relationships between BP measures and BMI in a South African adolescent cohort.

**Materials and methods:**

This longitudinal study (2010–2014) included 121 South African adolescent boys and girls of Black and White ethnicity from the Physical Activity Health Longitudinal Study. Measures included systolic blood pressure (SBP), diastolic blood pressure (DBP), pulse pressure, mid-blood pressure (Mid-BP), mean arterial pressure (MAP), and BMI.

**Results:**

Over four years, significant increases (*p* < 0.05) in BMI, SBP, DBP, PP, Mid-BP, and MAP were observed in adolescents (*p* < 0.001). BMI was consistently and positively associated with various BP measures across the study period. In 2012, BMI associated with SBP (*β* = 0.22; *p* = 0.018), DBP (*β* = 0.22; *p* = 0.018), Mid-BP (*β* = 0.24; *p* = 0.009), and MAP (*β* = 0.24; *p* = 0.009). These associations persisted in 2013, with stronger relationships observed for SBP (*β* = 0.27; *p* = 0.003), DBP (*β* = 0.21; *p* = 0.030), Mid-BP (*β* = 0.27; *p* = 0.004), and MAP (*β* = 0.27; *p* = 0.005). In 2014, BMI associated with Mid-BP (*β* = 0.22; *p* = 0.017) and MAP (*β* = 0.23; *p* = 0.015). After adjusting for the previous year's BP, BMI positively associated with SBP (*β* = 0.23; *p* = 0.013) in 2013 and DBP (*β* = 0.19; *p* = 0.049) in 2014. Significant associations remained between BMI and Mid-BP (*β* = 0.18–0.21; *p* = 0.022–0.047) and MAP (*β* = 0.19–0.20; *p* = 0.028–0.045) across 2012 and 2013.

**Conclusion:**

Cumulatively increasing BP significantly and positively associated with BMI, suggesting that increasing BMI may contribute to elevated BP during adolescence. Early identification and targeted lifestyle interventions are required to mitigate obesity-related elevated BP in South African adolescents.

## 1 Introduction

Adolescent obesity is a global health crisis, and low- and middle-income countries, including South Africa, carry the largest burden (1). Projections by World Obesity estimate that 27% of South African youth, 5–19 years of age, will suffer from obesity by 2030 (1). Obesity projections globally and in South African youth are worrisome as excess and dysfunctional adipose tissue results in poor health outcomes such as obesity-related elevated blood pressure (BP) (2).

Obesity is known as the primary risk factor for hypertension development in youth (3) and the severity of obesity-related hypertension is affected by the age of obesity onset (4). The process of obesity-related hypertension development begins from an early age as gestational weight gain is already positively associated with the offspring's BP (5). Obesity-related elevated BP in children and adolescents not only increases the risk of hypertension in adulthood but also associates with other adverse long-term cardiovascular health outcomes (3, 2), emphasizing the importance of advancing obesity-related BP research in youth.

In adolescence, mechanisms of longitudinal changes in obesity-related elevated BP are complex and various factors should be considered. Both systolic blood pressure (SBP) and diastolic blood pressure (DBP) increase with age, height (6), and puberty-related hormonal changes (2). Differences in obesity-related elevated BP between boys and girls also occur (2) and individuals of Black ethnicity are known to have an increased risk of developing hypertension when compared to individuals of White ethnicity (7). Lastly, mid-blood pressure (mid-BP) and mean arterial pressure (MAP) are thought to be better predictors of cardiovascular mortality when compared to SBP, DBP, and pulse pressure (PP) (8). Mean arterial pressure is also considered a superior predictor of obesity-related elevated BP when compared to SBP and DBP, respectively (9). It may therefore be beneficial to include Mid-BP and MAP when investigating the early development of obesity-related elevated BP.

Research regarding the relationship of various BP measures, including Mid-BP and MAP, with body mass index (BMI) in South African adolescents is scarce. This study aimed to investigate the longitudinal relationship between BMI and various BP measures in South African adolescent boys and girls of Black and White ethnicity to update and build on the body of health research in low- and middle-income countries.

## 2 Materials and methods

### 2.1 Study population

This study used longitudinal data from the Physical Activity Health Longitudinal Study (PAHLS), and a detailed description of the methodology was published elsewhere (10). The main aim of the PAHL study was to longitudinally investigate physical activity and its relationship with various health-related factors. Eight secondary schools, four from Potchefstroom Central town (high socio-economic status) and four from the Ikageng Township (low socio-economic status) of the Tlokwe Local Municipality, Potchefstroom, South Africa, were randomly selected to partake in this study. However, two schools from Potchefstroom Central town declined to take part in this study. This four-year (2010–2014) longitudinal study included 121 adolescents (*n* = 48 boys and *n* = 73 girls of *n* = 99 Black and *n* = 22 White ethnicity), with a median age of 14.08 (13.75; 14.42) years in 2010, from six schools of Potchefstroom, South Africa. Parents or guardians who regarded their child as healthy provided written informed consent and participants provided assent before participation. The PAHL study was approved by the Health Research Ethics Committee of the North-West University, Potchefstroom (NWU-0058-01-A1). Permission was granted by school principals and the Department of Basic Education of the North West Province, Potchefstroom, to conduct the study. This study complies with the Helsinki Declaration for medical research on human participants.

### 2.2 Anthropometric measurements

Anthropometric measurements were obtained by trained Anthropometrists according to standardized methods of the International Society for Advancement of Kinanthropometry (11). Body height was measured with a Harpenden portable stadiometer (Holtain Limited, Crymych, Dyfed, UK) to the nearest 0.1 cm, and body weight with an electronic scale (Beurer Ps07 Electronic Scale, Ulm, Germany) to the nearest 0.1 kg. Body mass index was calculated as weight (kg)/height (m^2^). Sex-specific BMI for age z-scores (BAZ), calculated with the World Health Organization 2007 *R Shiny* application Anthroplus software, were used to determine underweight or normal weight as BAZ ≤1 and overweight or obese (OW/OB) as BAZ >1 classification in adolescents according to the growth reference for children and adolescents 5–19 years old (12).

Blood pressure measurements were taken on the left arm using an Omron MIT Elite Plus (Omron Healthcare Co., Ltd, Japan) and an appropriately sized cuff. Participants were instructed to avoid stimulant beverages and rest in a seated position for five minutes before BP measurements were obtained. Talking was prohibited during the resting period and while BP measurements were taken. The average of two measurements, obtained at least five minutes apart, were used in the analysis. Blood pressure classification was calculated with the American Academy of Pediatrics 2017 *R Shiny* Application software from the Canadian Pediatric Endocrine Group, applicable to children 1–18 years old. The American Academy of Pediatrics clinical practice guidelines were used to classify adolescents who have elevated BP (≥90th) including hypertension (≥95th percentile) according to sex, age, and height-specific norms for SBP and/or DBP (6). Clinic PP was calculated as (SBP-DBP) and Mid-BP as the average of SBP and DBP (13). Clinic MAP was calculated as (DBP + 0.412 × PP), as it is considered a superior discriminator of target organ deterioration when compared to several other MAP estimates, including the traditional formula that uses the form factor value of 0.33 (14).

### 2.3 Statistical analysis

Data was analyzed with IBM® SPSS® Statistics Software version 29 (IBM Corporation, Armonk, New York) and graphical illustrations were created using GraphPad Prism 5. Descriptive statistics are expressed as arithmetic means with standard deviations, except for age which is presented as a median with 25th and 75th percentiles, and categorical data as proportions in percentage. Four-year longitudinal trends were determined using Repeated Analysis of Variance.

The longitudinal relationship between BP measures (SBP, DBP, PP, Mid-BP, and MAP) as dependent variables and BMI as the independent variable was determined using unadjusted Pearson correlations (Model A) and Partial correlations adjusted for age, sex, and ethnicity (Model B) at five cross-sectional time points for 2010, 2011, 2012, 2013, and 2014, respectively. Partial correlations were repeated with an additional adjustment for height (Model C). Multivariable analyses were used to further investigate the relationship between BP measures and BMI with adjustments for age, sex, ethnicity, and height in 2010, 2011, 2012, 2013, and 2014, respectively. Multivariable analyses were repeated with additional adjustments for the previous year's specific BP for each model in 2011, 2012, 2013, and 2014, respectively (i.e., the association between PP and BMI in 2011 was additionally adjusted for PP in 2010). Missing data was deleted in a pair-wise manner.

## 3 Results

Characteristics of the total group from the years 2010–2014 are illustrated in Table 1. The total group of 121 adolescents comprised 39.7% boys and 81.8% adolescents of Black ethnicity. Over four consecutive years, 21.5%, 21.5%, 22.3%, 19.0%, and 22.3% of adolescents were classified as OW/OB in 2010, 2011, 2012, 2013, and 2014, respectively. Elevated BP was prevalent in 19.6%, 10.9%, 15.1%, 23.1%, and 23.9% of adolescents in 2010, 2011, 2012, 2013, and 2014, respectively. Additionally, in Table 1, height, weight, BMI, SBP, DBP, PP, Mid-BP, and MAP increased significantly (*p* < 0.001) over four years.

**Table 1:** Characteristics of the total group from 2010 to 2014.

Total group = 121						
	2010	2011	2012	2013	2014	*p*-trend
Demographic information						
Age (years)	14.08 (13.75; 14.42)	15.00 (14.63; 15.29)	16.00 (15.63; 16.29)	17.00 (16.63; 17.33)	18.00 (17.63; 18.33)	**<0**.**001**
Sex, boys, *n* (%)	48 (39.7)	48 (39.7)	48 (39.7)	48 (39.7)	48 (39.7)	**–**
Ethnicity, Black *n* (%)	99 (81.8)	99 (81.8)	99 (81.8)	99 (81.8)	99 (81.8)	**–**
Anthropometric measures						
Height (m)	156.99 ± 8.07	160.34 ± 8.57	162.18 ± 8.09	163.60 ± 9.39	163.79 ± 9.70	**<0**.**001**
Weight (kg)	50.93 ± 12.29	54.70 ± 12.89	57.82 ± 13.32	59.96 ± 13.81	60.68 ± 14.29	**<0**.**001**
BMI (kg/m^2^)	20.51 ± 3.99	21.17 ± 4.22	21.90 ± 4.33	22.33 ± 4.45	22.55 ± 4.61	**<0**.**001**
BAZ, OW/OB, *n/N* (%)	26/121 (21.5)	26/121 (21.5)	27/121 (22.3)	23/121 (19.0)	27/121 (22.3)	**–**
Blood pressure measures						
SBP (mmHg)	104 ± 12	103 ± 10	106 ± 12	108 ± 10	115 ± 9	**<0**.**001**
DBP (mmHg)	65 ± 8	66 ± 6	66 ± 8	71 ± 8	73 ± 9	**<0**.**001**
PP (mmHg)	39 ± 12	36 ± 8	40 ± 9	37 ± 7	42 ± 10	**<0**.**001**
Mid-BP (mmHg)	85 ± 8	85 ± 7	86 ± 9	90 ± 9	94 ± 8	**<0**.**001**
MAP (mmHg)	81 ± 8	81 ± 7	83 ± 9	86 ± 8	90 ± 8	**<0**.**001**
Hypertensive, *n/N* (%)	18/92 (19.6)	11/101 (10.9)	18/119 (15.1)	27/117 (23.1)	28/117 (23.9)	**–**

Values are expressed as arithmetic means with standard deviations and proportions, whereas age is expressed as a median with 25th and 75th percentiles. Bold values denote statistical significance at *p* ≤ 0.05.

BAZ, body mass index for age; BMI, body mass index; DBP, diastolic blood pressure; MAP, mean arterial pressure; OW/OB, overweight or obese; PP, pulse pressure; and SBP, systolic blood pressure.

In the total group, unadjusted correlations (Model A) in Supplementary Table S1 showed significant positive correlations of SBP (*r* = 0.24; *p* = 0.010), DBP (*r* = 0.21; *p* = 0.025), Mid-BP (*r* = 0.25; *p* = 0.007), and MAP (*r* = 0.25; *p* = 0.008) with BMI in 2013. Partial correlations with adjustments for age, sex, and ethnicity (Model B) showed a significant positive correlation between SBP and BMI (*r* = 0.21; *p* = 0.047) in 2010, and significant positive correlations between SBP (*r* = 0.21; *p* = 0.022), DBP (*r* = 0.21; *p* = 0.021), Mid-BP (*r* = 0.24; *p* = 0.011), and MAP (*r* = 0.24; *p* = 0.011) in 2012. In the same model, SBP (*r* = 0.26; *p* = 0.005), DBP (*r* = 0.21; *p* = 0.029), Mid-BP (*r* = 0.26; *p* = 0.005), and MAP (*r* = 0.26; *p* = 0.006) correlated positively and significantly with BMI in 2013, and DBP (*r* = 0.22; *p* = 0.018), Mid-BP (*r* = 0.22; *p* = 0.018), and MAP (*r* = 0.22; *p* = 0.016) correlated positively and significantly with BMI in 2014. Partial correlations with adjustments for age, sex, ethnicity, and height (Model C) showed significant positive correlations of SBP (*r* = 0.22; *p* = 0.018), DBP (*r* = 0.22; *p* = 0.018), Mid-BP (*r* = 0.24; *p* = 0.009), and MAP (*r* = 0.24; *p* = 0.009) with BMI in 2012. In the same model, SBP (*r* = 0.28; *p* = 0.003), DBP (*r* = 0.20; *p* = 0.030), Mid-BP (*r* = 0.27; *p* = 0.004), and MAP (*r* = 0.26; *p* = 0.005) correlated positively and significantly with BMI in 2013, whereas DBP (*r* = 0.22; *p* = 0.021), Mid-BP (*r* = 0.22; *p* = 0.017), and MAP (*r* = 0.23; *p* = 0.015) correlated positively and significantly with BMI in 2014.

**Figure 1:**
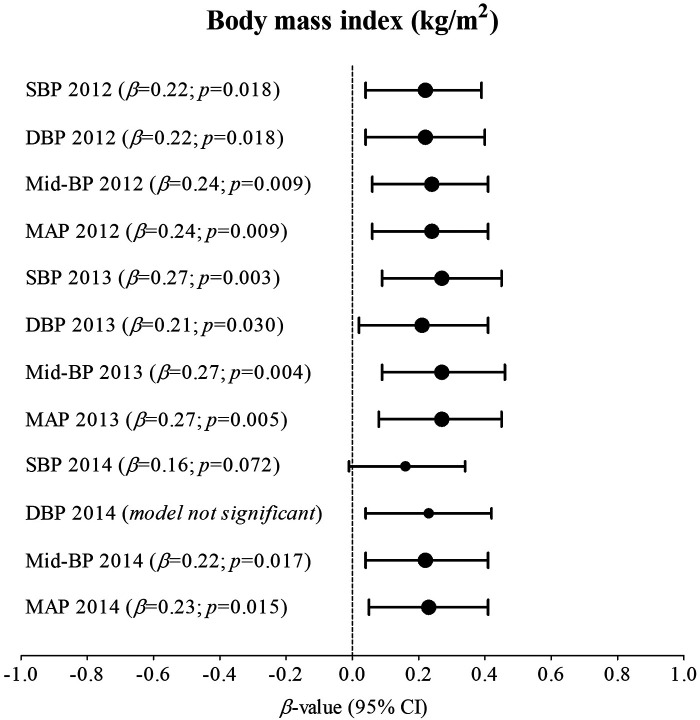
Associations between nblood pressure and body mass index in the total group with adjustments for age, sex, ethnicity, and height. Bold values denote significance (*p* < 0.05). CI, confidence intervals; diastolic blood pressure, DBP, mean arterial pressure, MAP, Mid-BP, mid-blood pressure, systolic blood pressure, SBP, and β, standardized beta.

**Figure 2:**
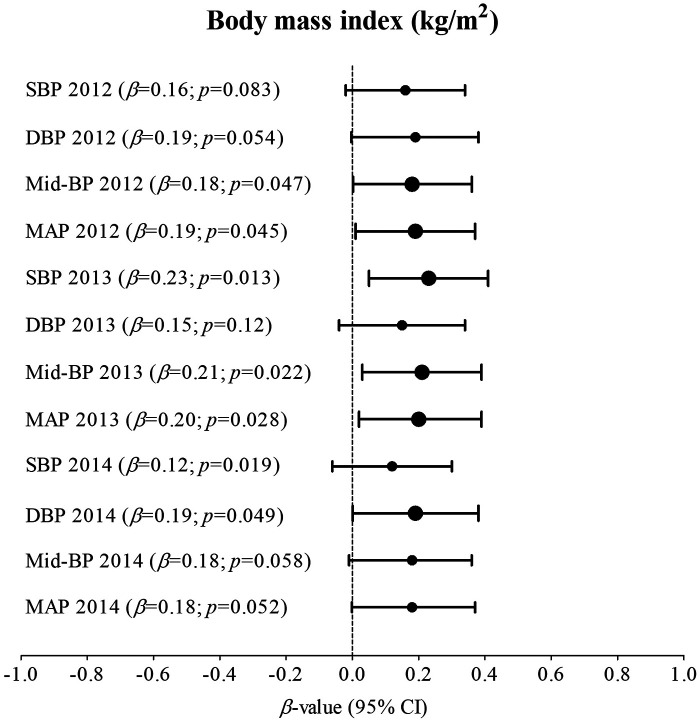
Associations between blood pressure and body mass index in the total group with adjustments for age, sex, ethnicity, height, and the previous year's blood pressure. Bold values denote significance (*p* < 0.05). CI, confidence intervals; diastolic blood pressure, DBP, mean arterial pressure, MAP, Mid-BP, mid-blood pressure, systolic blood pressure, SBP, and β, standardized beta.

Supplementary Table S2.1; Figure 1 indicates multivariable analysis between BP and BMI with adjustments for age, sex, ethnicity, and height in the total group. In 2012, SBP (adj. *R*^2^ = 0.17; *β* = 0.22; *p* = 0.018), DBP (adj. *R*^2^ = 0.14; *β* = 0.22; *p* = 0.018), Mid-BP (adj. *R*^2^ = 0.19; *β* = 0.24; *p* = 0.009), and MAP (adj. *R*^2^ = 0.19; *β* = 0.24; *p* = 0.009) associated positively and significantly with BMI. In 2013, SBP (adj. *R*^2^ = 0.18; *β* = 0.27; *p* = 0.003), DBP (adj. *R*^2^ = 0.06; *β* = 0.21; *p* = 0.030), Mid-BP (adj. *R*^2^ = 0.15; *β* = 0.27; *p* = 0.004), and MAP (adj. *R*^2^ = 0.14; *β* = 0.27; *p* = 0.005) associated positively and significantly with BMI. In 2014, Mid-BP (adj. *R*^2^ = 0.13; *β* = 0.22; *p* = 0.017), and MAP (adj. *R*^2^ = 0.11; *β* = 0.23; *p* = 0.015) associated positively and significantly with BMI in 2014. In Supplementary Table S2.2; Figure 2, the model was repeated with additional adjustments for the previous year's BP. In 2012, Mid-BP (adj. *R*^2^ = 0.31; *β* = 0.18; *p* = 0.047) and MAP (adj. *R*^2^ = 0.30; *β* = 0.19; *p* = 0.045) associated positively and significantly with BMI; whereas, SBP (adj. *R*^2^ = 0.23; *β* = 0.23; *p* = 0.013), Mid-BP (adj. *R*^2^ = 0.22; *β* = 0.21; *p* = 0.022), and MAP (adj. *R*^2^ = 0.21; *β* = 0.20; *p* = 0.028) associated positively and significantly with BMI in 2013. In 2014, DBP (adj. *R*^2^ = 0.11; *β* = 0.19; *p* = 0.049) associated positively and significantly with BMI.

## 4 Discussion

Body mass index and BP significantly increased over five-year measurement points (2010–2014) in adolescents, and significant positive associations were found of SBP and DBP with BMI in 2012 and 2013, and of Mid-BP and MAP with BMI in 2012, 2013, and 2014. In the same model of multivariable associations, we assessed the cumulative effect of obesity-related BP by additionally adjusting for the previous year's BP. The relationship of SBP and DBP with BMI lost significance in 2012 and the relationship between DBP and BMI lost significance in 2013. The relationship of Mid-BP and MAP with BMI also lost significance in 2014, but a weak positive relationship between DBP and BMI gained significance in 2014.

In this study, BMI significantly increased over four years in adolescents, and OW/OB classifications according to sex- and age-specific BMI z-scores showed a fluctuating increase with 21.5%, 21.5%, 22.3%, 19%, and 22.3% in 2010, 2011, 2012, 2013, and 2014, respectively. Blood pressure also significantly increased over four years. However, elevated BP according to age-, sex-, and height-specific z-scores showed a prevalence of 19.6% in 2010, dipping to 10.9% in 2011, whereafter it gradually increased to 15.1%, 23.1%, and 23.9% in 2012, 2013, and 2014, respectively. The higher BP in 2010 and dip in 2011 followed by a gradual yearly increase, is indicative of the “accommodation effect” in which participants adjust to the experience of repeated BP measurements (6). The gradual increases in BP after the “accommodation effect” has occurred may be explained by increases in BMI, among other factors (15).

In further analysis, partial correlations and multivariable associations showed significant positive relationships of SBP and DBP with BMI in 2012 and 2013, but not in 2014. Blood pressure, especially SBP, increases during the initial and mid-stages of puberty and reaches adult values at the end of puberty as a result of changes in growth hormones, insulin, and sex hormones (2). Moreover, growth hormones and insulin are amplified by obesity (2) and may partly explain the significant positive relationships of SBP and DBP with BMI in 2012 and 2013, but not in 2014 when height plateaued during the end of adolescence in our population.

In our study, Mid-BP and MAP showed significant positive relationships with BMI in 2012, 2013, and 2014. Mid-blood pressure and MAP are both defined as the average overall arterial pressure throughout a cardiac cycle (13). However, MAP is closer to DBP, also calculated as MAP = cardiac output × total peripheral resistance, and therefore largely determined by total peripheral resistance (16). Mechanisms regarding the relationship between MAP and BMI in our study may involve increased peripheral resistance as a result of microvascular dysfunction often observed in obesity (17). In fact, in children who suffer from obesity, microvascular dysfunction appears before advancing to macrovascular dysfunction in adulthood (18). Microvascular dysfunction is therefore thought to precede the development of hypertension (17), partly explaining our findings of an early positive relationship of MAP and Mid-BP with BMI in adolescents.

Another study in 1187 young South African men and women of Black and White ethnicity with a mean age of 24.6 ± 3.12 years compared the relationship between perfusion pressure and pulsatile pressure with subclinical target end-organ damage (19). Findings revealed that perfusion pressure measured by ambulatory MAP was more strongly associated with subclinical target end-organ damage when compared to pulsatile pressure measured by ambulatory PP (19). This suggests the superiority of perfusion pressure when investigating early cardiovascular disease development, especially in younger populations (19). However, more studies are needed to confirm that MAP and Mid-BP are more strongly related to subclinical target end-organ damage when compared to PP in overweight and obesogenic adolescents. Additionally, MAP and Mid-BP showed similar results in our study, although stronger relationships between obesity and MAP may occur as microvascular function deteriorates over time, but further investigations are needed.

In this study, we repeated multivariable associations between BP and BMI with an additional adjustment for the previous year's BP to take the cumulative effects of BP into account (20). The significant positive relationships of BMI with Mid-BP and MAP in 2012 and 2013, and of SBP with BMI in 2013 remained, but as previously mentioned, this may be partly explained by hormonal changes during puberty (2). In 2014, the relationship of Mid-BP and MAP with BMI lost significance, emphasizing the cumulative effect of obesity-related BP in adolescents and the need to identify and implement targeted educational lifestyle therapies for obesity-related elevated BP from an early age.

We also found a weak, but significant positive association between DBP and BMI in 2014 after additional adjustments for the previous year's BP. Recent research focusing on the relationship between obesity and DBP in South African adolescents is scanty. However, another study in 8475 Chinese patients with primary hypertension investigated the different risk factors for isolated systolic hypertension and isolated diastolic hypertension across different age groups >18 years of age (21). Isolated diastolic hypertension was more prevalent in age groups 18–64 years and associated with a higher BMI and smoking whereas isolated systolic hypertension was more prevalent in age groups >65 years and associated with older age (21). The relationship between DBP and BMI in younger populations is in line with our results. It was previously mentioned that increased DBP in obesity may be the product of increased left ventricular end-diastolic pressure (22). Additionally, DBP is directly proportional to total peripheral resistance (16), which again is linked to microvascular dysfunction often found in obesity (17). Detailed mechanisms by which obesity increases BP are beyond the scope of this manuscript, but also may involve inflammation, oxidative stress, vascular dysfunction (23), increased plasma volume, sympathetic nervous system overactivity, and hyperinsulinemia (24).

Additional factors considered to contribute to changes in BP during adolescence involve age, sex, ethnicity, and height, or changes during puberty. A pooled meta-analysis and systematic review confirmed that age and BMI are positively related to increases in SBP and DBP in 37926 sub-Saharan African adolescents aged 10–19 years (25). It should however be noted that BP increases with age as a result of an increase in body size during adolescence, and not necessarily as a result of the ageing process (2), and height should therefore be considered in addition to chronological age (6). Adolescent sex differences in BP should also be considered, as girls generally have lower BP when compared to boys, which is primarily attributed to a lower stroke volume and lower total peripheral resistance (2). This is a result of estrogen in girls, which increases nitric oxide-induced vasorelaxation (2) and drives subcutaneous adipose tissue distribution, protecting girls against the accumulation of visceral adipose tissue (26). Regarding ethnic differences in BP, individuals of Black ethnicity are known to have an increased risk of developing hypertension when compared to individuals of White ethnicity (7). We therefore adjusted for age, sex, ethnicity, and height when investigating the relationship between BP and BMI in further analysis as the size of our study population did not allow for a split in sex or ethnicity.

Taken together, our results indicate early relationships of SBP, DBP, Mid-BP, and MAP with BMI in South African adolescents. Our results suggest that the relationships between SBP, DBP, and BMI may be amplified during the mid-stages of adolescence and reach a plateau closer to the end of adolescence, despite our adjustments for age and height. After height has plateaued in our population, perfusion pressure measured by Mid-BP and MAP positively and significantly related to BMI and should be considered alongside other BP measures when investigating cardiometabolic health in adolescents, until more research is done. In addition, the cumulative effects of obesity-related BP are already present in this South African cohort of adolescents, which emphasizes the need for early identification and targeted educational lifestyle interventions to mitigate obesity-related elevated BP in South African youth.

This paper should be viewed within its strengths and limitations. We included adolescent boys and girls of Black and White ethnicity. This population did not allow for a split into groups according to sex, ethnicity, or normal- and overweight or obese groups due to insufficient statistical power, but we did adjusted for sex and ethnicity. Significant relationships were moderate-to-weak, but strong relationships are not expected in such a young cohort which also included lean adolescents. This study also used office BP, and not ambulatory BP, which may have underestimated the number of individuals who suffer from elevated BP. This may also underestimate the strength of associations between BP and obesity as masked hypertension, measured by ambulatory BP monitoring, is especially present in adolescents who suffer from obesity or are of Black ethnicity (7). We acknowledge that obesity cannot be defined only by BMI; however, superior measures require advanced assessments and equipment which are not always available to low- and middle-income countries (1), such as South Africa. Longitudinal and cross-sectional data were used for this study and causality could therefore not be established, but our results may contribute to the body of literature on early identification of cardiometabolic disease in South African adolescents. Additionally, drop-out during the study period could be attributed to factors beyond our control, such as illness or absenteeism, which is a common issue in South African schools on the day of data collection and the emigration of participants’ families from the study areas to other regions due to employment opportunities.

The authors of this study conclude that during mid-adolescence, the relationship of BMI with SBP and DBP are amplified, but Mid-BP and MAP remain related to BMI after height reaches a plateau at the end stages of adolescence in this South African cohort. Obesity-related BP has a cumulative effect, highlighting the need for early identification and targeted educational lifestyle interventions to mitigate obesity-related elevated BP in South African adolescents.

## Data Availability

The original contributions presented in the study are included in the article/Supplementary Material, further inquiries can be directed to the corresponding author.
