# Supplementary material for: Longitudinal blood pressure and body mass index in South African adolescents
Source: Front Pediatr. 2025 Sep 8;13:1643812. doi: 10.3389/fped.2025.1643812 (PMC12450704; doi:10.3389/fped.2025.1643812)
Supplement: Supplementary file 1 [file Table1.docx]

Supplementary Material

**Supplementary Table 1.** Correlations of blood pressure with body mass index in the total group.

|  | **Body mass index (kg/m^2^)** | | | | | |
| --- | --- | --- | --- | --- | --- | --- |
|  | ***Total group (n=121)*** | | | | | |
|  | **Model A** | | **Model B** | | **Model C** | |
|  | ***r*** | ***p*-value** | ***r*** | ***p*-value** | ***r*** | ***p*-value** |
| ***2010 (n=92)*** |  |  |  |  |  |  |
| Systolic blood pressure (mmHg) | 0.18 | 0.088 | **0.21** | **0.047** | 0.18 | 0.086 |
| Diastolic blood pressure (mmHg) | -0.001 | 0.99 | 0.05 | 0.66 | 0.04 | 0.72 |
| Pulse pressure (mmHg) | 0.18 | 0.078 | 0.19 | 0.083 | 0.16 | 0.13 |
| Mid-blood pressure (mmHg) | 0.12 | 0.24 | 0.17 | 0.10 | 0.15 | 0.16 |
| Mean arterial pressure (mmHg) | 0.11 | 0.32 | 0.16 | 0.14 | 0.14 | 0.21 |
| ***2011 (n=101)*** |  |  |  |  |  |  |
| Systolic blood pressure (mmHg) | 0.07 | 0.51 | 0.18 | 0.083 | 0.17 | 0.099 |
| Diastolic blood pressure (mmHg) | 0.01 | 0.89 | 0.12 | 0.25 | 0.11 | 0.27 |
| Pulse pressure (mmHg) | 0.07 | 0.50 | 0.10 | 0.32 | 0.10 | 0.35 |
| Mid-blood pressure (mmHg) | 0.05 | 0.63 | 0.17 | 0.093 | 0.16 | 0.11 |
| Mean arterial pressure (mmHg) | 0.04 | 0.67 | 0.17 | 0.10 | 0.16 | 0.12 |
| ***2012 (n=119)*** |  |  |  |  |  |  |
| Systolic blood pressure (mmHg) | 0.15 | 0.11 | **0.21** | **0.022** | **0.22** | **0.018** |
| Diastolic blood pressure (mmHg) | 0.14 | 0.14 | **0.21** | **0.021** | **0.22** | **0.018** |
| Pulse pressure (mmHg) | 0.06 | 0.50 | 0.07 | 0.47 | 0.07 | 0.46 |
| Mid-blood pressure (mmHg) | 0.15 | 0.094 | **0.24** | **0.011** | **0.24** | **0.009** |
| Mean arterial pressure (mmHg) | 0.15 | 0.095 | **0.24** | **0.011** | **0.24** | **0.009** |
| ***2013 (n=117)*** |  |  |  |  |  |  |
| Systolic blood pressure (mmHg) | **0.24** | **0.010** | **0.26** | **0.005** | **0.28** | **0.003** |
| Diastolic blood pressure (mmHg) | **0.21** | **0.025** | **0.21** | **0.029** | **0.20** | **0.030** |
| Pulse pressure (mmHg) | 0.08 | 0.42 | 0.09 | 0.33 | 0.11 | 0.25 |
| Mid-blood pressure (mmHg) | **0.25** | **0.007** | **0.26** | **0.005** | **0.27** | **0.004** |
| Mean arterial pressure (mmHg) | **0.25** | **0.008** | **0.26** | **0.006** | **0.26** | **0.005** |
| ***2014 (n=120)*** |  |  |  |  |  |  |
| Systolic blood pressure (mmHg) | 0.06 | 0.49 | 0.16 | 0.094 | 0.17 | 0.072 |
| Diastolic blood pressure (mmHg) | 0.13 | 0.14 | **0.22** | **0.018** | **0.22** | **0.021** |
| Pulse pressure (mmHg) | -0.06 | 0.51 | -0.06 | 0.52 | -0.05 | 0.62 |
| Mid-blood pressure (mmHg) | 0.11 | 0.22 | **0.22** | **0.018** | **0.22** | **0.017** |
| Mean arterial pressure (mmHg) | 0.12 | 0.19 | **0.22** | **0.016** | **0.23** | **0.015** |

Model A: unadjusted; Model B: adjusted for age, sex, and ethnicity; Model C: adjusted for age, sex, ethnicity, and height. Bold values denote statistically significant correlations (*p*<0.05).

**Supplementary Table 2.1.** Multivariable analysis between blood pressure and body mass index in the total group.

|  | **Body mass index (kg/m^2^)** | | |
| --- | --- | --- | --- |
|  | ***Total group (n=121)*** | | |
|  | Adj. R^2^ | *β* (95% CI) | *p*-value |
| ***2010 (n=92)*** |  |  |  |
| Systolic Blood Pressure (mmHg) | NS | NS | NS |
| Diastolic Blood Pressure (mmHg) | 0.10 | 0.04 (-0.17; 0.25) | 0.72 |
| Pulse Pressure (mmHg) | NS | NS | NS |
| Mid-Blood Pressure (mmHg) | 0.08 | 0.15 (-0.06; 0.37) | 0.16 |
| Mean Arterial Pressure (mmHg) | 0.09 | 0.14 (-0.08; 0.35) | 0.21 |
| ***2011 (n=101)*** |  |  |  |
| Systolic Blood Pressure (mmHg) | 0.11 | 0.17 (-0.03; 0.37) | 0.099 |
| Diastolic Blood Pressure (mmHg) | 0.09 | 0.11 (-0.09; 0.32) | 0.27 |
| Pulse Pressure (mmHg) | NS | NS | NS |
| Mid-Blood Pressure (mmHg) | 0.14 | 0.16 (-0.04; 0.36) | 0.11 |
| Mean Arterial Pressure (mmHg) | 0.13 | 0.16 (-0.04; 0.35) | 0.12 |
| ***2012 (n=119)*** |  |  |  |
| Systolic Blood Pressure (mmHg) | **0.17** | **0.22 (0.04; 0.39)** | **0.018** |
| Diastolic Blood Pressure (mmHg) | **0.14** | **0.22 (0.04; 0.40)** | **0.018** |
| Pulse Pressure (mmHg) | NS | NS | NS |
| Mid-Blood Pressure (mmHg) | **0.19** | **0.24 (0.06; 0.41)** | **0.009** |
| Mean Arterial Pressure (mmHg) | **0.19** | **0.24 (0.06; 0.41)** | **0.009** |
| ***2013 (n=117)*** |  |  |  |
| Systolic Blood Pressure (mmHg) | **0.18** | **0.27 (0.09; 0.45)** | **0.003** |
| Diastolic Blood Pressure (mmHg) | **0.06** | **0.21 (0.02; 0.41)** | **0.030** |
| Pulse Pressure (mmHg) | NS | NS | NS |
| Mid-Blood Pressure (mmHg) | **0.15** | **0.27 (0.09; 0.46)** | **0.004** |
| Mean Arterial Pressure (mmHg) | **0.14** | **0.27 (0.08; 0.45)** | **0.005** |
| ***2014 (n=120)*** |  |  |  |
| Systolic Blood Pressure (mmHg) | 0.19 | 0.16 (-0.01; 0.34) | 0.072 |
| Diastolic Blood Pressure (mmHg) | NS | NS | NS |
| Pulse Pressure (mmHg) | 0.11 | -0.05 (-0.23; 0.14) | 0.62 |
| Mid-Blood Pressure (mmHg) | **0.13** | **0.22 (0.04; 0.41)** | **0.017** |
| Mean Arterial Pressure (mmHg) | **0.11** | **0.23 (0.05; 0.41)** | **0.015** |

Bold values denote significance (*p*<0.05). Multivariable analysis was adjusted for age, sex, ethnicity, and height. *Abbreviations: Adj. R^2^, adjusted R-square; CI, confidence intervals; NS, model not significant; and Std. β, standardized beta.*

**Supplementary Table 2.2.** Multivariable analysis between blood pressure and body mass index in the total group with additional adjustments for the previous year’s blood pressure.

|  | **Body mass index (kg/m^2^)** | | |
| --- | --- | --- | --- |
|  | ***Total group (n=121)*** | | |
|  | Adj. R^2^ | *β* (95% CI) | *p*-value |
| ***2010 (n=92)*** |  |  |  |
| Systolic Blood Pressure (mmHg) | - | - | - |
| Diastolic Blood Pressure (mmHg) | - | - | - |
| Pulse Pressure (mmHg) | - | - | - |
| Mid-Blood Pressure (mmHg) | - | - | - |
| Mean Arterial Pressure (mmHg) | - | - | - |
| ***2011 (n=101)*** |  |  |  |
| Systolic Blood Pressure (mmHg) | 0.20 | 0.09 (-0.14; 0.32) | 0.46 |
| Diastolic Blood Pressure (mmHg) | NS | NS | NS |
| Pulse Pressure (mmHg) | NS | NS | NS |
| Mid-Blood Pressure (mmHg) | 0.26 | 0.09 (-0.13; 0.31) | 0.43 |
| Mean Arterial Pressure (mmHg) | 0.24 | 0.09 (-0.13; 0.32) | 0.40 |
| ***2012 (n=119)*** |  |  |  |
| Systolic Blood Pressure (mmHg) | 0.29 | 0.16 (-0.02; 0.34) | 0.083 |
| Diastolic Blood Pressure (mmHg) | 0.20 | 0.19 (-0.003; 0.38) | 0.054 |
| Pulse Pressure (mmHg) | NS | NS | NS |
| Mid-Blood Pressure (mmHg) | **0.31** | **0.18 (0.002; 0.36)** | **0.047** |
| Mean Arterial Pressure (mmHg) | **0.30** | **0.19 (0.01; 0.37)** | **0.045** |
| ***2013 (n=117)*** |  |  |  |
| Systolic Blood Pressure (mmHg) | **0.23** | **0.23 (0.05; 0.41)** | **0.013** |
| Diastolic Blood Pressure (mmHg) | 0.14 | 0.15 (-0.04; 0.34) | 0.12 |
| Pulse Pressure (mmHg) | 0.07 | 0.11 (-0.09; 0.30) | 0.28 |
| Mid-Blood Pressure (mmHg) | **0.22** | **0.21 (0.03; 0.39)** | **0.022** |
| Mean Arterial Pressure (mmHg) | **0.21** | **0.20 (0.02; 0.39)** | **0.028** |
| ***2014 (n=120)*** |  |  |  |
| Systolic Blood Pressure (mmHg) | 0.22 | 0.12 (-0.06; 0.30) | 0.19 |
| Diastolic Blood Pressure (mmHg) | **0.11** | **0.19 (0.001; 0.38)** | **0.049** |
| Pulse Pressure (mmHg) | 0.16 | -0.07 (-0.25; 0.11) | 0.45 |
| Mid-Blood Pressure (mmHg) | 0.18 | 0.18 (-0.01; 0.36) | 0.058 |
| Mean Arterial Pressure (mmHg) | 0.16 | 0.18 (-0.001; 0.37) | 0.052 |

Bold values denote significance (*p*<0.05). Multivariable analysis was adjusted for age, sex, ethnicity, height, and the previous year’s blood pressure. *Abbreviations: Adj. R^2^, adjusted R-square; CI, confidence intervals; NS, model not significant; and Std. β, standardized beta.*
